# A patient survey on the impact of alkaptonuria symptoms as perceived by the patients and their experiences of receiving diagnosis and care

**DOI:** 10.1002/jmd2.12101

**Published:** 2020-03-07

**Authors:** Mattias Rudebeck, Ciarán Scott, Nicolas Sireau, Lakshminarayan Ranganath

**Affiliations:** ^1^ Swedish Orphan Biovitrum Stockholm Sweden; ^2^ AKU Society Cambridge UK; ^3^ Department of Clinical Biochemistry & Metabolic Medicine Royal Liverpool University Hospital Liverpool UK

**Keywords:** Alkaptonuria, impact of symptoms, orphan disease, patient experience, quality of life, rare disease

## Abstract

**Background:**

Alkaptonuria (AKU) is an ultrarare and multifaceted disease characterized by the absence of functional homogentisate 1,2‐dioxygenase activity, the enzyme responsible for breakdown of homogentisic acid—a tyrosine‐degradation product. The presymptomatic phase of the disease makes diagnosis difficult, with many patients unidentified or diagnosed late in life.

**Objective:**

To date, no study has analyzed the perceived impact of different symptoms or the experiences of individuals through the patient journey in the context of AKU. This study aimed to examine patients' perceptions of AKU symptoms and their impact on quality of life as well as patients' experiences of being diagnosed and living with the disease.

**Methods:**

Data for this study were collected using a quantitative self‐report questionnaire administered online to people with AKU.

**Results:**

Data from 45 participants indicate that symptoms with the highest impact for patients are those related to pain and ruptures, disability and inability to perform normal routines, emotional/mental health issues, and heart complications. Findings also revealed significant delays in contact with healthcare services and time to diagnosis. Furthermore, patients reported difficulty in receiving information about AKU, treatment and care, and long‐term disease management support.

**Conclusions:**

Time to diagnosis and care of AKU is significantly delayed. Symptoms of AKU with the highest impact on quality of life for patients are those related to pain and disability and the inability to perform normal routines. Bridging any gaps between patients with AKU and healthcare professionals through education could help improve patients' experiences with AKU through the patient journey.

**SYNOPSIS**

Identifying initiatives to improve patient and healthcare professional (HCP) knowledge is of utmost importance to not only accelerate diagnosis but also to improve patient's quality of life and harmonize patient‐HCP interactions.

## 1 INTRODUCTION

Alkaptonuria (AKU) is an ultrarare disease, described by Archibald Garrod as the prototype for proving his theory of “inborn errors of metabolism” during his Croonian lectures of 1908.^1^, ^2^ AKU is characterized by the absence of functional homogentisate 1,2‐dioxygenase activity, the enzyme responsible for the breakdown of a tyrosine‐degradation product—homogentisic acid (HGA).^3^ The resulting accumulation of HGA is oxidized to a melanin‐like pigment in a process known as ochronosis which results in blue/black pigment in the eye, skin, and connective tissue, especially cartilage. The term was introduced in 1866 for the observation of black cartilage in a patient postmortem.^4^ AKU is a multifaceted disease, with symptoms affecting individuals differently. Other features of AKU include arthritis, joint destruction, aortic stenosis, vascular calcifications, and dark urine due to the presence of HGA in the urine.^5^, ^6^, ^7^, ^8^

The estimated incidence of AKU is 1:250 000 to 1 000 000.^7^ A rare disease has been defined as having a prevalence of <1/2000 whereas an ultrarare disease affects <1/50 000 people.^9^ While AKU falls into the latter, it is most commonly reported in India, Jordan, and Slovakia, and analysis of affected families suggests the high incidence in such areas may be due to a loss of genetic variation.^2^

There is currently no pharmacological treatment available, and treatment options are limited to management of the disease sequelae as they arise, including physiotherapy, surgery, and analgesia. Ascorbic acid, low protein diets, and lifestyle counseling are just some of the therapeutic approaches that have been tried with little to no success due to lack of evidence or unproven efficacy.^10^ A current development program is investigating nitisinone (also known as NTBC) as a potential treatment for AKU (NCT01916382^11^, ^12^). Nitisinone inhibits 4‐hydroxyphenylpyruvate, the enzyme responsible for the production of HGA, and is currently approved for the treatment of hereditary tyrosinaemia type 1.^6^, ^10^

AKU is a slowly progressing disease with a presymptomatic phase that makes diagnosis difficult. Many patients are also misdiagnosed with the more common osteoarthritis.^13^, ^14^ Dark urine and staining of nappies are the earliest clues for diagnosing AKU and it can be confirmed by urine HGA measurement.^6^, ^8^ However, these clues are often missed, only to become apparent later in adult life when signs and symptoms appear.^10^, ^13^ Delay in patient referral as well as the high number of misdiagnosed patients are common factors associated with diagnostic delay, and a lack of patient awareness (not associating symptoms with the disease) means patients usually seek healthcare services after the therapeutic “window of opportunity.”^15^ Providing individuals and families with relevant disease information through genetic counseling can help them make more informed medical and personal decisions,^6^ and therefore it is imperative that the disease‐specific diagnostic journey is understood by healthcare professionals (HCPs) and that they unite with patients to create a society of trust and collaboration^16^ and optimal support.

To date, no study has analyzed the patients' perceived impact of symptoms on quality of life or the experiences of individuals through the patient journey in the context of AKU, and since these patients may experience unmet needs, a study was performed using a quantitative, patients' self‐reported online questionnaire. The study aimed to (a) examine patients' perceptions of the impact that AKU symptoms have on quality of life, and (b) examine aspects of patients' experiences of being diagnosed and living with the disease in relation to receiving adequate support and care.

## 2 METHODS

### 2.1 Sample

#### 2.1.1 Participants

A total of 120 invitations to participate in the electronic survey were distributed to individuals connected to and registered in the database of the AKU Society, United Kingdom, and their international sister organizations in the United Kingdom, France, Italy, the Netherlands, Slovakia, Jordan, and Spain. People with a confirmed diagnosis of AKU and consent to participate in the survey were eligible for inclusion. No other inclusion or exclusion criteria were applied.

#### 2.1.2 Ethical procedure

The protocol was notified to relevant ethics review boards if required by local regulations for this type of research. Confirmed informed consent was obtained from all participants in the electronic form before any data collection. The invitation included information to the participants and a link to the survey. The invitees were informed that by clicking the link they consented to participate in the study and confirmed they had read the survey information without any further unanswered questions. Before entering the data collection questionnaire, they also had to click a statement confirming “I have read the survey information provided to me and consent to participate in the survey.”

### 2.2 Survey

#### 2.2.1 Quantitative research

The self‐report questionnaire comprised of 16 items, covering the following areas:

- Socio‐demographic and disease‐related information: Patients were asked to specify their age category, sex, and experienced symptoms of the disease.
- Quality of life‐related information: Participants were asked to indicate their perceived impact of each symptom whether they had already experienced these symptoms, or in the event they would experience them in the future. Each symptom's impact was assessed as “not sure,” “not at all,” “not very high,” “fairly,” “very high,” or “extremely high.”
- Time to medical care from initial symptoms.
- Time to diagnosis from initial medical care.
- Number of physicians visited before patient received diagnosis.
- Reasons for any delayed contact with medical care from initial symptoms (all relevant options that applied should be given).
- Incorrect initial diagnosis was measured by asking participants to indicate “yes,” “no,” or “not sure.”
- Patients' perceptions of sufficient knowledge of AKU (ie, the individual's feeling of their own level of knowledge of the disease) was measured by asking participants to indicate “yes,” “no,” or “not sure.”
- Patients' main source of disease information: Participants were asked to identify their key sources for disease information (all relevant options that applied should be given).
- Patients' perceptions of HCPs' sufficient knowledge of AKU by asking participants to indicate “yes,” “no,” or “not sure.”
- Patients' experiences in interactions with HCPs to diagnosis and care: how easy/difficult was it for patients to interact with HCPs across multiple circumstances. Participants were asked to indicate “not sure,” “very easy,” “somewhat easy,” “neither easy nor difficult,” “somewhat difficult,” or “very difficult.”

#### 2.2.2 Delivery

The questionnaire was delivered online using the Typeform (Typeform S.L., Barcelona) survey tool and was translated into the official language of all participating countries. Translations were performed by forward translations and confirmatory reviews were performed by native speakers of the target languages.

#### 2.2.3 Data handling and analysis

The AKU Society, UK, handled all data during the study, including storage, source documentation, and quality control of the final analysis file and of completeness of data collection to assess usability of data. Descriptive analysis was conducted by total sample, country, age category, and sex.

## 3 RESULTS

### 3.1 Sample characteristics

A total of 45 participants, 23 males (51.1%) and 22 females (48.9%), completed the survey (a 37.5% response rate), with the majority residing in the United Kingdom (31.1%), France (17.8%), and Slovakia (22.2%). The demographic and clinical characteristics are shown in Table S1. Visual pigmentation (82.2%), lower back pain (88.9%), joint pain (95.6%), and stiffness (84.4%) were the most common symptoms experienced before participating in the survey, with the majority of participants aged between 40 and 74 years **(** Table S1 **)**.

### 3.2 Perceived impact of symptoms

**Figure 1:**
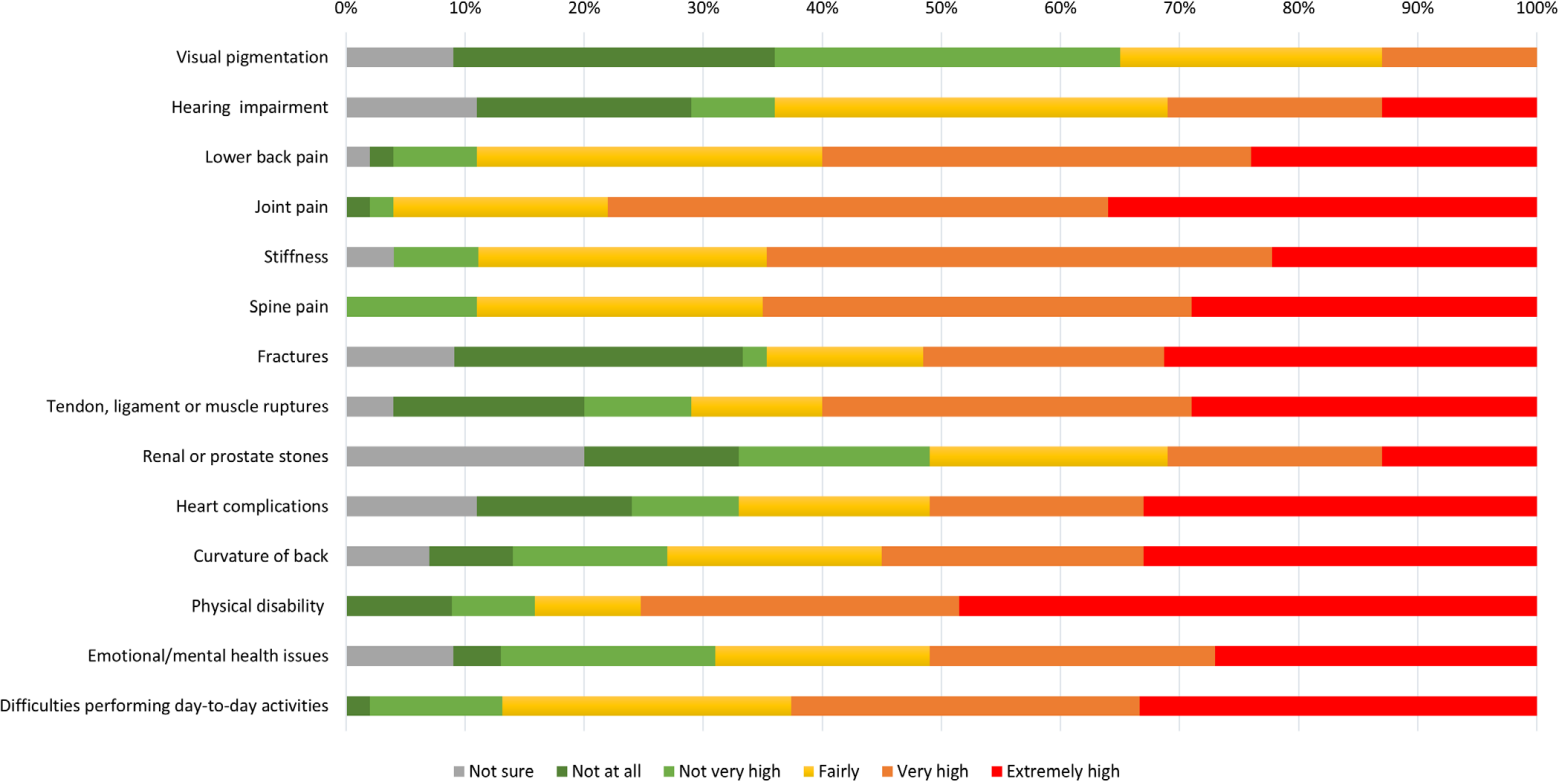
Patients' perceived impact of AKU symptoms, all patients (N = 45)

The symptoms with the highest impact and outcomes assessed by >50% of the patients as “extremely high” and “very high” combined were joint pain (78%), physical disability (76%), spine pain (65%), stiffness (64%), difficulties in performing daily activities (62%), lower back pain (60%), tendon/ligament/muscle ruptures (60%), curvature of back (56%), emotional/mental health issues (51%), fractures (51%), and heart complications (51%) **(**Figure 1 **)**.

The majority of participants rated the impact of visual pigmentation as “not at all” or “not very high” (56%), while impact of hearing impairment and renal/prostate stones was evenly assessed across the lower (“not at all” and “not very high”), medium (“fairly”), and higher (“very high” and “extremely high”) ratings **(**Figure 1 **)**.

Furthermore, female participants reported a higher impact compared to male participants for the following symptoms: physical disability, emotional/mental health issues, difficulties in performing daily activities, joint pain, heart complications, visual pigmentation, and hearing impairment **(** Table S2 **)**.

### 3.3 Receiving diagnosis and care

Overall, contact with healthcare services from initial symptoms was delayed to 9.9 years (mean), with later contact being made by male patients (11.2 years [mean]) compared to females (8.7 years [mean]). Additionally, time to medical care was delayed to 1.7 years (mean) in Slovakia (n = 6), compared to a mean of 13.0 and 10.7 years in the United Kingdom (n = 8) and France (n = 8), respectively **(**Table 1 **)**.

**Table 1:** Time to diagnosis from medical care, number of physicians visited before receiving diagnosis, and incorrect initial diagnosis

	Time to medical care from initial symptoms (years): mean (min, max) N = 30 [n]	Time to diagnosis from medical care (years): mean (min, max) N = 26 [n]	Number of physicians visited before diagnosis: mean (min, max) N = 34 [n]	Incorrect initial diagnosis; N = 45 [n]			
				No	Yes	Not sure	
Overall population	9.94 (0, 58.75) [30]	4.07 (0, 37.56) [26]	3.0 (1, 10) [34]	40.0% [18]	46.7% [21]	13.3% [6]
Sex						
Male	11.22 (0, 58.75) [15]	3.25 (0, 37.56) [13]	3.1 (1, 10) [17]			
Female	8.65 (0, 50.32) [15]	4.88 (0, 21.10) [13]	2.8 (1, 6) [17]			
Country						
United Kingdom	13.03 (0, 53.70) [8]	8.15 (0, 37.56) [7]	3.4 (1, 10) [9]	35.7% [5]	57.1% [8]	7.1% [1]
France	10.73 (0.04, 58.75) [8]	3.86 (0, 21.10) [8]	4.0 (1, 10) [7]	12.5% [1]	62.5% [5]	25.0% [2]
Italy	20.73 (12.68, 28.78) [2]	15.26 (15.26, 15.26) [1]	2.0 (1, 3) [2]	100.0% [3]	0% [0]	0% [0]
The Netherlands	16.80 (0, 50.32) [3]	0.46 (0, 0.92) [2]	2.5 (1, 4) [4]	25.0% [1]	50.0% [2]	25.0% [1]
Slovakia	1.74 (0, 10.43) [6]	0.11 (0, 0.50) [7]	2.3 (1, 5) [8]	70.0% [7]	20.0% [2]	10.0% [1]
Jordan	2.83 (2.83, 2.83) [1]		3.0 (1, 5) [2]	0% [0]	75.0% [3]	25.0% [1]
Spain	1.50 (0, 3.00) [2]	0.75 (0.75, 0.75) [1]	2 (2, 2) [2]	50.0% [1]	50.0% [1]	0% [0]

Abbreviations: min, minimum; max, maximum.

Following contact with healthcare services, time to diagnosis was also delayed (4.1 years [mean]), with male patients being diagnosed earlier than females (3.3 years [mean] and 4.9 years [mean], respectively), while on average, three physicians were consulted prior to receiving a diagnosis **(**Table 1 **)**.

A concerning number of patients indicated that not interpreting the signs as symptoms of the disease (40.0%) and not considering symptoms to be severe enough (26.7%) were the main reasons for not seeking medical care earlier from initial symptoms. In addition, 17.8% of patients were unsure where to seek medical care or had other reasons for delaying contact with healthcare services (Table 2 **)**. Of the 45 patients participating in the survey, almost half (n = 21; 46.7%) were initially diagnosed incorrectly across the participating countries **(**Table 1 **)**.

**Table 2:** Reasons for delayed contact with medical care from initial symptoms

Population	Reasons for delayed contact with medical care from initial symptoms^a^									
	Symptoms not severe enough	Did not interpret the signs as symptoms of disease	Thought symptoms could be self‐managed	Thought symptoms would go away without medical care	Afraid to not be taken seriously by HCP	Do not like going to a physician	Concerned about the cost	Unsure where to seek medical care	Patient/caregiver too busy	Not sure/other
	% (n)	% (n)	% (n)	% (n)	% (n)	% (n)	% (n)	% (n)	% (n)	% (n)
Overall (N = 45)	26.7 (12)	40.0 (18)	15.6 (7)	13.3 (6)	6.7 (3)	0 (0)	4.4 (2)	17.8 (8)	4.4 (2)	17.8 (8)
Sex										
Male (n = 23)	17.4 (4)	26.1 (6)	8.7 (2)	8.7 (2)	4.3 (1)	0 (0)	4.3 (1)	21.7 (5)	4.3 (1)	30.4 (7)
Female (n = 22)	36.4 (8)	54.5 (12)	22.7 (5)	18.2 (4)	9.1 (2)	0 (0)	4.5 (1)	13.6 (3)	4.5 (1)	4.5 (1)
Country										
UK (n = 14)	14.3 (2)	35.7 (5)	7.1 (1)	14.3 (2)	0 (0)	0 (0)	0 (0)	7.1 (1)	0 (0)	35.7 (5)
France (n = 8)	37.5 (3)	50.0 (4)	12.5 (1)	12.5 (1)	12.5 (1)	0 (0)	0 (0)	12.5 (1)	25.0 (2)	12.5 (1)
Italy (n = 3)	66.7 (2)	0 (0)	33.3 (1)	0 (0)	0 (0)	0 (0)	0 (0)	0 (0)	0 (0)	0 (0)
The Netherlands (n = 4)	50.0 (2)	25.0 (1)	25.0 (1)	0 (0)	0 (0)	0 (0)	0(0)	50.0 (2)	0 (0)	0 (0)
Slovakia (n = 10)	20.0 (2)	50.0 (5)	20.0 (2)	20.0 (2)	10.0 (1)	0 (0)	0 (0)	20.0 (2)	0 (0)	20.0 (2)
Jordan (n = 4)	0 (0)	25.0 (1)	0 (0)	0 (0)	25.0 (1)	0 (0)	25.0 (1)	50.0 (2)	0 (0)	0 (0)
Spain (n = 2)	50.0 (1)	100.0 (2)	50.0 (1)	50.0 (1)	0 (0)	0 (0)	50.0 (1)	0 (0)	0 (0)	0 (0)

a Multiple responses could be given.

### 3.4 Knowledge of disease

A large number of patients (n = 33; 73.3%) felt they themselves had sufficient knowledge of AKU **(** Table S3 **)**. Almost half of the patients (n = 22; 48.9%) considered HCPs to not have sufficient knowledge of the disease **(** Table S4 **)**.

The quantitative research also found that HCPs (57.8%) and patient organizations (57.8%) were the main sources for patients accessing disease information, with a reasonable number of patients indicating they accessed information via the internet (35.6%) and literature (42.2%) **(** Table S5 **)**.

### 3.5 Experiences of interactions with HCPs

**Figure 2:**
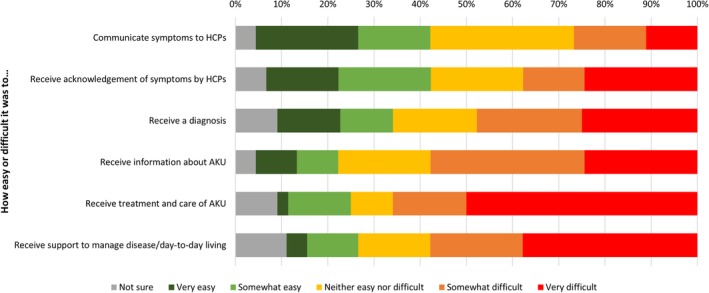
Patients' experiences in interactions with HCPs regarding diagnosis and care, all patients (N = 45)

The interactions with the highest difficulty assessed by the patients as “somewhat difficult” and “difficult” combined were receiving information about AKU (57.8%), receiving treatment and care of AKU (62.2%), and disease management support (57.8%) across all participating countries. On the other hand, those aspects of interacting with HCPs which indicated a less burden for patients as assessed by responses with highest reporting of “very easy” or “easy” combined were regarding the communication of symptoms (33.3%) and receiving acknowledgement of symptoms (35.6%; Figure 2 and Table S6).

Receiving a diagnosis from HCPs was reported to be more difficult among female patients compared to males, while more male patients reported difficulty in receiving treatment and care of AKU (Table S6).

## 4 DISCUSSION

This quantitative survey study among patients with AKU provided valuable insight into how patients with AKU experience living with the disease. Data indicate that symptoms with the highest impact for patients were those related to pain and ruptures, disability and inability to perform normal routines, emotional/mental health issues, and heart complications. While the majority of participants rated the impact of visual pigmentation as “not at all” or “not very high,” pigmentation in the eyes and ears reflect burden of pigment throughout the body and is the major pathophysiology in AKU which leads to tissue damage.^17^ It is vital that HCPs be informed of these findings to optimize the management and improve the quality of life of these patients. Therapeutic alternatives are lacking for the disease. Current treatment approaches remain palliative and focus on pain control, physiotherapy, and joint replacement. Gene and enzyme replacement therapies are being investigated, although development of such therapies is complex.^2^ A potential pharmacological treatment is also currently being investigated.^10^

Findings from this study revealed significant delays in contact with healthcare services and time to diagnosis. While many patients often forget they are diagnosed as infants, many visit HCPs later in life when symptoms appear, but follow‐up prior to this is lost and may explain the delay in seeking medical care. Also, the high number of misdiagnosed patients further contributes to delays in diagnosis and optimal care. Organizational changes in healthcare and increasing social awareness of rare diseases and AKU are just two initiatives that may help limit diagnostic delays.^15^ Furthermore, improving patient and HCP knowledge of AKU can help accelerate the diagnostic process by ensuring patients understand the severity of their symptoms and when to seek medical care, while also ensuring HCPs make a definitive diagnosis earlier in the patient journey. As some polymorphisms may not be disease‐causing, molecular genetic testing is still not the gold standard for the diagnosis of AKU and accessing genetic testing is a common challenge encountered by HCPs.^18^ Therefore, urine HGA measurement, as of now, remains the classical diagnostic approach.^6^, ^8^, ^10^

A large number of patients felt they had sufficient knowledge themselves of AKU, especially in countries that have centers treating a large number of patients (France, the United Kingdom, and Slovakia), which may be a contributing factor. However, participants indicated that patient organizations served as a vital community for receiving disease information. It is essential that these communities continue to provide peer‐to‐peer support and information on AKU, encouraging new connections and raising awareness to not only improve the patient journey but patient quality of life. In addition, a reasonable number of patients reported the internet as a source for obtaining disease information, and with the rise of healthcare in social media,^19^ patients have access to a digital platform full of educational resources, thus allowing patients to independently make a suggestive diagnosis via a simple search term, for example, “black urine.”

In interactions with HCPs, participants reported difficulty in receiving information about AKU, treatment and care, and long‐term disease management support. The absence of major registries is an issue for rare diseases, including AKU, as patients are not tracked and records may be lost.^20^ Strategies in the UK are in place to set up a national registry for AKU in the near future that can help track patients after diagnosis, characterize populations, and identify target groups for intervention.^13^

This quantitative study had a few limitations. Being an online survey without direct interactions with the participants may introduce an increased risk of individual interpretation of questions; therefore, the findings of this study should be interpreted bearing this in mind. Despite being a survey study, the response rate of 37.5% was considered a satisfactory figure in the field of rare diseases. A previous survey identifying individuals with AKU targeted primary‐care physicians (n = 11 151) via postal delivery and obtained a response rate of 18.2%.^13^ Using an online survey tool is considered to have aided the logistics of survey administration.^21^ An element of bias or patient misreporting must also be considered in the context of patients' perceptions of disease knowledge. Almost half of the patients (48.9%) considered HCPs did not have sufficient knowledge of AKU, although 57.8% reported that HCPs were a key source for accessing disease information. Additionally, the survey was only conducted in countries where AKU perception and knowledge is already high within the disease community.

For many families, general practitioners (GPs) are the first point of contact; however, their awareness and recognition of AKU are lacking. Several initiatives are in place to empower both patients and GPs, including a training module with the Royal College of General Practitioners on how to diagnose AKU and a variety of downloadable resources that can be shared with GPs and patients at first diagnosis. Furthermore, the AKU Society attend GP national meetings and deliver information on AKU, while also serving the opportunity to work with GPs and be available at the first point of patients' symptoms, to not only empower patient groups to work with GPs, but for patients to work with each other within the patient groups.

While this is the first study analyzing the perceived impact of symptoms of AKU, further research is warranted. The AKU Society now has possession of a survey that can be distributed further to gain additional input and data over time, and repeated sampling may help improve generalizability of study results.

Data from this survey provide information on how people with a rare disease, AKU, experience living with the condition and may help bridge gaps between patients and HCPs, which in turn may have a positive impact on patients' experiences with AKU through the patient journey. Accelerating the diagnosis of AKU is of utmost importance and findings from this survey may help identify initiatives to improve patient and HCP knowledge. Furthermore, while the presence of centers with significant clinical experience of AKU in Slovakia, France, and the United Kingdom offers effective management and monitoring of the disease, data from this survey may help provide support not only to countries where these centers are absent, but to countries where an early identification program is absent. Findings from this study may also help put existing and upcoming clinical trial results such as SONIA 2 (NCT01916382) and SOFIA^22^ in the perspective of the patient to further assess aspects of clinical relevance of such results.

## CONFLICT OF INTEREST

Rudebeck is an employee and shareholder of Sobi. Scott, Sireau, and Ranganath have no competing interests to disclose.

## AUTHOR CONTRIBUTIONS

M.R.: study design, conduct of the study, data analysis, and writing of the manuscript. C.S.: conduct of the study, data analysis, and writing of the manuscript. N.S.: study design, data analysis, and writing of the manuscript. L.R.: study design, data analysis, and writing of the manuscript. All authors gave final approval of the version to be published.

## ETHICAL APPROVAL STATEMENT

The protocol was notified to relevant ethics review boards if required by local regulations for this type of research.

## INFORMED CONSENT

Informed consent was obtained from all participants prior to inclusion in the study.

## Supporting information


**Appendix** **S1:** Supporting information
