# Supplementary material for: A patient survey on the impact of alkaptonuria symptoms as perceived by the patients and their experiences of receiving diagnosis and care
Source: JIMD Rep. 2020 Mar 7;53(1):71–9. doi: 10.1002/jmd2.12101 (PMC7203644; doi:10.1002/jmd2.12101)

**Table 1. Demographic characteristics per age group**

|  |  | | |  |  |  |  |
| --- | --- | --- | --- | --- | --- | --- | --- |
| **Age group** | **Under 18 years**  **n (%) N=1** | **18–25 years**  **n (%) N=2** | **26–39 years**  **n (%) N=5** | **40–59 years**  **n (%) N=18** | **60–74 years**  **n (%) N=15** | **Above 75 years**  **n (%) N=4** | **Total**  **n (%)**  **N=45** |
| **Sex** | | | |  |  |  |  |
| Male | 1 (100.0%) | 1 (50.0%) | 4 (80.0%) | 12 (66.7%) | 5 (33.3%) | 0 (0%) | 23 (51.1%) |
| Female | 0 (0%) | 1 (50.0%) | 1 (20.0%) | 6 (33.3%) | 10 (66.7%) | 4 (100.0%) | 22 (48.9%) |
| **Country** | | | |  |  |  |  |
| United Kingdom | 0 (0%) | 0 (0%) | 1 (20.0%) | 5 (27.8%) | 7 (46.7%) | 1 (25.0%) | 14 (31.1%) |
| France | 0 (0%) | 0 (0%) | 0 (0%) | 3 (16.7%) | 4 (26.7%) | 1 (25.0%) | 8 (17.8%) |
| Italy | 0 (0%) | 0 (0%) | 0 (0%) | 2 (11.1%) | 0 (0%) | 1 (25.0%) | 3 (6.7%) |
| Netherlands | 0 (0%) | 0 (0%) | 0 (0%) | 2 (11.1%) | 1 (6.7%) | 1 (25.0%) | 4 (8.9%) |
| Slovakia | 0 (0%) | 2 (100.0%) | 3 (60.0%) | 2 (11.1%) | 3 (20.0%) | 0 (0%) | 10 (22.2%) |
| Jordan | 1 (100.0%) | 0 (0%) | 1 (20.0%) | 2 (11.1%) | 0 (0%) | 0 (0%) | 4 (8.9%) |
| Spain | 0 (0%) | 0 (0%) | 0 (0%) | 2 (11.1%) | 0 (0%) | 0 (0%) | 2 (4.4%) |
| **Symptoms of alkaptonuria experienced^a^** | | | |  |  |  |  |
| Visual pigmentation | 0 (0%) | 1 (50.0%) | 2 (40.0%) | 15 (83.3%) | 15 (100.0%) | 4 (100.0%) | 37 (82.2%) |
| Hearing impairment | 0 (0%) | 1 (50.0%) | 1 (20.0%) | 1 (5.6%) | 5 (33.3%) | 1 (25.0%) | 9 (20.0%) |
| Lower back pain | 1 (100.0%) | 2 (100.0%) | 4 (80.0%) | 17 (94.4%) | 14 (93.3%) | 2 (50.0%) | 40 (88.9%) |
| Joint pain | 1 (100.0%) | 2 (100.0%) | 4 (80.0%) | 17 (94.4%) | 15 (100.0%) | 4 (100.0%) | 43 (95.6%) |
| Stiffness | 1 (100.0%) | 2 (100.0%) | 1 (20.0%) | 15 (83.3%) | 15 (100.0%) | 4 (100.0%) | 38 (84.4%) |
| Spine pain | 1 (100.0%) | 2 (100.0%) | 2 (40.0%) | 16 (88.9%) | 12 (80.0%) | 2 (50.0%) | 35 (77.8%) |
| Fractures | 0 (0%) | 0 (0%) | 1 (20.0%) | 2 (11.1%) | 3 (20.0%) | 1 (25.0%) | 7 (15.6%) |
| Tendon, ligament or muscle ruptures | 1 (100.0%) | 0 (0%) | 0 (0%) | 7 (38.9%) | 8 (53.3%) | 3 (75.0%) | 19 (42.2%) |
| Renal or prostate stones | 0 (0%) | 0 (0%) | 1 (20.0%) | 6 (33.3%) | 2 (13.3%) | 1 (25.0%) | 10 (22.2%) |
| Heart complications | 0 (0%) | 0 (0%) | 2 (40.0%) | 2 (11.1%) | 0 (0%) | 2 (50.0%) | 6 (13.3%) |
| Curvature of back | 1 (100.0%) | 1 (50.0%) | 0 (0%) | 9 (50.0%) | 8 (53.3%) | 1 (25.0%) | 20 (44.4%) |
| Physical disability | 0 (0%) | 0 (0%) | 0 (0%) | 11 (61.1%) | 7 (46.7%) | 2 (50.0%) | 20 (44.4%) |
| Emotional/mental health issues | 1 (100.0%) | 1 (50.0%) | 1 (20.0%) | 9 (50.0%) | 8 (53.3%) | 2 (50.0%) | 22 (48.9%) |
| Difficulties performing day-to-day activities | 0 (0%) | 0 (0%) | 0 (0%) | 9 (50.0%) | 7 (46.7%) | 0 (0%) | 16 (35.6%) |
| None | 0 (0%) | 0 (0%) | 0 (0%) | 0 (0%) | 0 (0%) | 0 (0%) | 0 (%) |
| Other | 0 (0%) | 0 (0%) | 0 (0%) | 0 (0%) | 0 (0%) | 0 (0%) | 0 (%) |
|  | | | |  |  |  |  |

^a^ multiple responses could be given

Table 2. Perceived impact of symptoms of alkaptonuria per sex

|  |  | |
| --- | --- | --- |
| **Sex** | **Male**  **n (%) N=23** | **Female**  **n (%)**  **N=22** |
| **Perceived impact of symptoms of alkaptonuria** | | |
| **Visual pigmentation** |  |  |
| Not sure | 4 (17.4%) | 0 (0%) |
| Not at all | 7 (30.4%) | 5 (22.7%) |
| Not very high | 7 (30.4%) | 6 (27.3%) |
| Fairly | 5 (21.7%) | 5 (22.7%) |
| Very high | 0 (0%) | 6 (27.3%) |
| Extremely high | 0 (0%) | 0 (0%) |
| **Hearing impairment** |  |  |
| Not sure | 4 (17.4%) | 1 (4.5%) |
| Not at all | 4 (17.4%) | 4 (18.2%) |
| Not very high | 2 (8.7%) | 1 (4.5%) |
| Fairly | 7 (30.4%) | 8 (36.4%) |
| Very high | 2 (8.7%) | 6 (27.3%) |
| Extremely high | 4 (17.4%) | 2 (9.1%) |
| **Lower back pain** |  |  |
| Not sure | 1 (4.3%) | 0 (0%) |
| Not at all | 1 (4.3%) | 0 (0%) |
| Not very high | 0 (0%) | 3 (13.6%) |
| Fairly | 7 (30.4%) | 6 (27.3%) |
| Very high | 10 (43.5%) | 6 (27.3%) |
| Extremely high | 4 (17.4%) | 7 (31.8%) |
| **Joint pain** |  |  |
| Not sure | 0 (0%) | 0 (0%) |
| Not at all | 1 (4.3%) | 0 (0%) |
| Not very high | 1 (4.3%) | 0 (0%) |
| Fairly | 5 (21.7%) | 3 (13.6%) |
| Very high | 11 (47.8%) | 8 (36.4%) |
| Extremely high | 5 (21.7%) | 11 (50.0%) |
| **Stiffness** |  |  |
| Not sure | 2 (8.7%) | 0 (0%) |
| Not at all | 0 (0%) | 0 (0%) |
| Not very high | 1 (4.3%) | 2 (9.1%) |
| Fairly | 5 (21.7%) | 6 (27.3%) |
| Very high | 12 (52.2%) | 7 (31.8%) |
| Extremely high | 3 (13.0%) | 7 (31.8%) |
| **Spine pain** |  |  |
| Not sure | 0 (0%) | 0 (0%) |
| Not at all | 0 (0%) | 0 (0%) |
| Not very high | 3 (13.0%) | 2 (9.1%) |
| Fairly | 5 (21.7%) | 6 (27.3%) |
| Very high | 10 (43.5%) | 6 (27.3%) |
| Extremely high | 5 (21.7%) | 8 (36.4%) |
| **Fractures** |  |  |
| Not sure | 0 (0%) | 4 (18.2%) |
| Not at all | 8 (34.8%) | 3 (13.6%) |
| Not very high | 1 (4.3%) | 0 (0%) |
| Fairly | 3 (13.0%) | 3 (13.6%) |
| Very high | 4 (17.4%) | 5 (22.7%) |
| Extremely high | 7 (30.4%) | 7 (31.8%) |
| **Tendon, ligament or muscle ruptures** |  |  |
| Not sure | 0 (0%) | 2 (9.1%) |
| Not at all | 4 (17.4%) | 3 (13.6%) |
| Not very high | 3 (13.0%) | 1 (4.5%) |
| Fairly | 4 (17.4%) | 1 (4.5%) |
| Very high | 5 (21.7%) | 9 (40.9%) |
| Extremely high | 7 (30.4%) | 6 (27.3%) |
| **Renal or prostate stones** |  |  |
| Not sure | 3 (13.0%) | 6 (27.3%) |
| Not at all | 3 (13.0%) | 3 (13.6%) |
| Not very high | 6 (26.1%) | 1 (4.5%) |
| Fairly | 3 (13.0%) | 6 (27.3%) |
| Very high | 6 (26.1%) | 2 (9.1%) |
| Extremely high | 2 (8.7%) | 4 (18.2%) |
| **Heart complications** |  |  |
| Not sure | 3 (13.0%) | 2 (9.1%) |
| Not at all | 5 (21.7%) | 1 (4.5%) |
| Not very high | 2 (8.7%) | 2 (9.1%) |
| Fairly | 4 (17.4%) | 3 (13.6%) |
| Very high | 5 (21.7%) | 3 (13.6%) |
| Extremely high | 4 (17.4%) | 11 (50.0%) |
| **Curvature of back** |  |  |
| Not sure | 2 (8.7%) | 1 (4.5%) |
| Not at all | 3 (13.0%) | 0 (0%) |
| Not very high | 5 (21.7%) | 1 (4.5%) |
| Fairly | 3 (13.0%) | 5 (22.7%) |
| Very high | 3 (13.0%) | 7 (31.8%) |
| Extremely high | 7 (30.4%) | 8 (36.4%) |
| **Physical disability** |  |  |
| Not sure | 0 (0%) | 0 (0%) |
| Not at all | 4 (17.4%) | 0 (0%) |
| Not very high | 2 (8.7%) | 1 (4.5%) |
| Fairly | 3 (13.0%) | 1 (4.5%) |
| Very high | 6 (26.1%) | 6 (27.3%) |
| Extremely high | 8 (34.8%) | 14 (63.6%) |
| **Emotional/mental health issues** |  |  |
| Not sure | 2 (8.7%) | 2 (9.1%) |
| Not at all | 2 (8.7%) | 0 (0%) |
| Not very high | 7 (30.4%) | 1 (4.5%) |
| Fairly | 3 (13.0%) | 5 (22.7%) |
| Very high | 6 (26.1%) | 5 (22.7%) |
| Extremely high | 3 (13.0%) | 9 (40.9%) |
| **Difficulties performing day-to-day activities** |  |  |
| Not sure | 0 (0%) | 0 (0%) |
| Not at all | 1 (4.3%) | 0 (0%) |
| Not very high | 4 (17.4%) | 1 (4.5%) |
| Fairly | 7 (30.4%) | 4 (18.2%) |
| Very high | 6 (26.1%) | 7 (31.8%) |
| Extremely high | 5 (21.7%) | 10 (45.5%) |
|  | | |

Table 3. Patients’ perceptions of their own knowledge of AKU

|  |  | |  | |  |  |
| --- | --- | --- | --- | --- | --- | --- |
|  | **Sufficient knowledge of disease**  **n (%)** | **Not sufficient knowledge of disease**  **n (%)** | **Not sure**  **n (%)** | |  | |
| **Overall population (N=45)** | | | |  | |  |
|  | 33 (73.3%) | 7 (15.6%) | 5 (11.1%) | |  | |
| **Sex** | | | |  | |  |
| Male (n=23) | 17 (73.9%) | 3 (13.0%) | 3 (13.0%) | |  | |
| Female (n=22) | 16 (72.7%) | 4 (18.2%) | 2 (9.1%) | |  | |
| **Country** | | | |  | |  |
| United Kingdom (n=14) | 12 (85.7%) | 2 (14.3%) | 0 (0%) | |  | |
| France (n=8) | 5 (62.5%) | 3 (37.5%) | 0 (0%) | |  | |
| Italy (n=3) | 2 (66.7%) | 0 (0%) | 1 (33.3%) | |  | |
| Netherlands (n=4) | 3 (75.0%) | 1 (25.0%) | 0 (0%) | |  | |
| Slovakia (n=10) | 7 (70.0%) | 1 (10.0%) | 2 (20.0%) | |  | |
| Jordan (n=4) | 3 (75.0%) | 0 (0%) | 1 (25.0%) | |  | |
| Spain (n=2) | 1 (50.0%) | 0 (0%) | 1 (50.0%) | |  | |
|  | | | |  | |  |

Table 4. Patients’ perceptions of healthcare professionals’ knowledge of AKU

|  |  | | |  | |  |
| --- | --- | --- | --- | --- | --- | --- |
|  | **Sufficient knowledge of disease**  **n (%)** | **Not sufficient knowledge of disease**  **n (%)** | **Not sure**  **n (%)** | |  | |
| **Overall population (N=45)** | | | |  | |  |
|  | 19 (42.2%) | 22 (48.9%) | 4 (8.9%) | |  | |
| **Country** | | | |  | |  |
| United Kingdom (n=14) | 6 (42.9%) | 8 (57.1%) | 0 (0%) | |  | |
| France (n=8) | 0 (0%) | 8 (100.0%) | 0 (0%) | |  | |
| Italy (n=3) | 0 (0%) | 1 (33.3%) | 2 (66.7%) | |  | |
| Netherlands (n=4) | 2 (50.0%) | 2 (50.0%) | 0 (0%) | |  | |
| Slovakia (n=10) | 6 (60.0%) | 2 (20.0%) | 2 (20.0%) | |  | |
| Jordan (n=4) | 3 (75.0%) | 1 (25.0%) | 0 (0%) | |  | |
| Spain (n=2) | 2 (100.0%) | 0 (0%) | 0 (0%) | |  | |
|  | | | |  | |  |

Table 5. Patients’ main source of disease information^a^

|  |  | | |  |  |  |  |
| --- | --- | --- | --- | --- | --- | --- | --- |
|  | **HCPs**  **n (%)** | **Literature**  **n (%)** | **Patient organisations**  **n (%)** | **Internet**  **n (%)** | **Not sure**  **n (%)** | **Other**  **n (%)** |  |
| **Overall population (N=45)** | | | |  |  |  |  |
|  | 26 (57.8%) | 19 (42.2%) | 26 (57.8%) | 16 (35.6%) | 2 (4.4%) | 4 (8.9%) |  |
| **Sex** | | | |  |  |  |  |
| Male (n=23) | 14 (60.9%) | 10 (43.5%) | 11 (47.8%) | 7 (30.4%) | 2 (8.7%) | 2 (8.7%) |  |
| Female (n=22) | 12 (54.5%) | 9 (40.9%) | 15 (68.2%) | 9 (40.9%) | 0 (0%) | 2 (9.1%) |  |
| **Country** | | | |  |  |  |  |
| United Kingdom (n=14) | 7 (50.0%) | 8 (57.1%) | 10 (71.4%) | 2 (14.3%) | 0 (0%) | 0 (0%) |  |
| France (n=8) | 4 (50.0%) | 4 (50.0%) | 8 (100.0%) | 4 (50.0%) | 0 (0%) | 2 (25.0%) |  |
| Italy (n=3) | 1 (33.3%) | 0 (0%) | 1 (33.3%) | 2 (66.7%) | 0 (0%) | 0 (0%) |  |
| Netherlands (n=4) | 3 (75.0%) | 3 (75.0%) | 2 (50.0%) | 1 (25.0%) | 0 (0%) | 1 (25.0%) |  |
| Slovakia (n=10) | 5 (50.0%) | 3 (30.0%) | 4 (40.0%) | 5 (50.0%) | 0 (0%) | 1 (10.0%) |  |
| Jordan (n=4) | 4 (100.0%) | 1 (25.0%) | 1 (25.0%) | 2 (50.0%) | 2 (50.0%) | 0 (0%) |  |
| Spain (n=2) | 2 (100.0%) | 0 (0%) | 0 (0%) | 0 (0%) | 0 (0%) | 0 (0%) |  |
| HCP, healthcare professional | | | |  |  |  |  |

^a^ multiple responses could be given

Table 6. Patients’ experiences in interactions with healthcare professionals to diagnosis and care per sex

|  |  | |  |
| --- | --- | --- | --- |
| Sex | Male  n (%) N=23 | Female  n (%)  N=22 |  |
| **How easy/difficult to…?** | | |  |
| Communicate their symptoms to HCPs |  |  |  |
| Not sure | 1 (4.3%) | 1 (4.5%) |  |
| Very easy | 5 (21.7%) | 5 (22.7%) |  |
| Somewhat easy | 5 (21.7%) | 0 (0%) |  |
| Neither easy nor difficult | 5 (21.7%) | 9 (40.9%) |  |
| Somewhat difficult | 5 (21.7%) | 4 (18.2%) |  |
| Very difficult | 2 (8.7%) | 3 (13.6%) |  |
| Receive acknowledgement of symptoms by HCPs |  |  |  |
| Not sure | 2 (8.7%) | 1 (4.5%) |  |
| Very easy | 3 (13.0%) | 4 (18.2%) |  |
| Somewhat easy | 6 (26.1%) | 3 (13.6%) |  |
| Neither easy nor difficult | 4 (17.4%) | 5 (22.7%) |  |
| Somewhat difficult | 3 (13.0%) | 3 (13.6%) |  |
| Very difficult | 5 (21.7%) | 6 (27.3%) |  |
| Receive a diagnosis |  |  |  |
| Not sure | 3 (13.0%) | 1 (4.5%)* |  |
| Very easy | 4 (17.4%) | 2 (9.1%)* |  |
| Somewhat easy | 3 (13.0%) | 2 (9.1%)* |  |
| Neither easy nor difficult | 6 (26.1%) | 2 (9.1%)* |  |
| Somewhat difficult | 5 (21.7%) | 5 (22.7%)* |  |
| Very difficult | 2 (8.7%) | 9 (40.9%)* |  |
| Receive information about AKU |  |  |  |
| Not sure | 1 (4.3%) | 1 (4.5%) |  |
| Very easy | 3 (13.0%) | 1 (4.5%) |  |
| Somewhat easy | 3 (13.0%) | 1 (4.5%) |  |
| Neither easy nor difficult | 4 (17.4%) | 5 (22.7%) |  |
| Somewhat difficult | 7 (30.4%) | 8 (36.4%) |  |
| Very difficult | 5 (21.7%) | 6 (27.3%) |  |
| Receive treatment and care of AKU |  |  |  |
| Not sure | 1 (4.3%) | 3 (13.6%)* |  |
| Very easy | 1 (4.3%) | 0 (0%)* |  |
| Somewhat easy | 3 (13.0%) | 3 (13.6%)* |  |
| Neither easy nor difficult | 2 (8.7%) | 2 (9.1%)* |  |
| Somewhat difficult | 4 (17.4%) | 3 (13.6%)* |  |
| Very difficult | 12 (52.2%) | 10 (45.5%)* |  |
| Receive support to manage disease/day-to-day living |  |  |  |
| Not sure | 3 (13.0%) | 2 (9.1%) |  |
| Very easy | 2 (8.7%) | 0 (0%) |  |
| Somewhat easy | 2 (8.7%) | 3 (13.6%) |  |
| Neither easy nor difficult | 4 (17.4%) | 3 (13.6%) |  |
| Somewhat difficult | 6 (26.1%) | 3 (13.6%) |  |
| Very difficult | 6 (26.1%) | 11 (50.0%) |  |
| AKU, alkaptonuria; HCP, healthcare professional *1 missing response | | |  |

**Supplementary data: Questionnaire**

⃝ I have read the survey information provided to me and consent to participate in the survey.

| **Which of the following categories best describes your age?** | | | | | |
| --- | --- | --- | --- | --- | --- |
| ⃝ Under 18 | ⃝ 18-25 | ⃝ 26-39 | ⃝ 40-59 | ⃝ 60-74 | ⃝ 75+ |

| **Gender** | |
| --- | --- |
| ⃝ Male | ⃝ Female |

| **Have you been diagnosed with alkaptonuria by a physician?** |
| --- |
| ⃝ No/Not sure *STOP -* *only diagnosed patients are eligible to complete the survey   Thank you for your interest!* |
| ⃝ Yes *Please continue to fill in the entire survey* |

| **Please indicate any of the below symptoms/medical events of alkaptonuria you have experienced (select as many as applicable)** |
| --- |
| ⃝ Visual pigmentation (e.g. dark spots on eyes or ears etc.) |
| ⃝ Hearing impairment |
| ⃝ Lower back pain |
| ⃝ Joint pain |
| ⃝ Stiffness |
| ⃝ Spine pain |
| ⃝ Fractures |
| ⃝ Tendon, ligament or muscle ruptures |
| ⃝ Renal or prostate stones |
| ⃝ Heart complications |
| ⃝ Curvature of the back (kyphosis or scoliosis) |
| ⃝ Physical disability (loss of motion or movement) |
| ⃝ Emotional/mental health issues (e.g. fatigue, depression, anxiety, limitations in social activities) |
| ⃝ Difficulties in performing normal routines/day-to-day activities |
| ⃝ Other _______________________________ |
| ⃝ None of these |

| **Below are listed a number of potential symptoms of alkaptonuria. If you are or were to be affected by these in the future, how would you perceive the impact of each of these on the overall quality of your life?** | | | | | | |
| --- | --- | --- | --- | --- | --- | --- |
|  | **Not at all** | **Not very high** | **Fairly** | **Very high** | **Extremely high** | **Not sure** |
| **Visual pigmentation (e.g. on eyes or ears)** |  |  |  |  |  |  |
| **Hearing impairment** |  |  |  |  |  |  |
| **Lower back pain** |  |  |  |  |  |  |
| **Joint pain** |  |  |  |  |  |  |
| **Stiffness** |  |  |  |  |  |  |
| **Spine pain** |  |  |  |  |  |  |
| **Fractures** |  |  |  |  |  |  |
| **Tendon, ligament or muscle ruptures** |  |  |  |  |  |  |
| **Renal or prostate stones** |  |  |  |  |  |  |
| **Heart complications** |  |  |  |  |  |  |
| **Curvature of the back (kyphosis or scoliosis)** |  |  |  |  |  |  |
| **Physical disability (loss of motion or movement)** |  |  |  |  |  |  |
| **Emotional/mental health issues (e.g. fatigue, depression, anxiety, limitations in social activities)** |  |  |  |  |  |  |
| **Difficulties in performing normal routines/day-to-day activities** |  |  |  |  |  |  |

| **When were your first symptoms of alkaptonuria noticed?** | |
| --- | --- |
| ⃝ ⃝ ⃝ ⃝ ⃝ ⃝ ⃝ ⃝ |  |
| D D M M Y Y Y Y | |

| **When did you or your caregiver seek medical care after your first symptoms were noticed?** | | |  |
| --- | --- | --- | --- |
| ⃝ ⃝ ⃝ ⃝ ⃝ ⃝ ⃝ ⃝ |  |  |  |
| D D M M Y Y Y Y | | |  |
| **If you (or your caregiver) waited before seeking medical care, were any of the below a reason for this (select as many as applicable)?** | | | |
| ⃝ Not applicable, I saw a physician almost immediately | | | |
| ⃝ The symptoms were not severe enough | | | |
| ⃝ Did not interpret the signs as symptoms of a disease | | | |
| ⃝ Thought the symptoms could be self-managed | | | |
| ⃝ Thought the symptoms would go away without medical care | | | |
| ⃝ Was afraid not to be taken seriously by health care professionals | | | |
| ⃝ Do not like going to a physician or receiving treatments | | | |
| ⃝ Was concerned about the cost of seeking medical care | | | |
| ⃝ Was unsure where to seek medical care for the kind of symptoms experienced | | | |
| ⃝ You/your caregiver was too busy | | | |
| ⃝ Not sure / Other | | | |

| **After first seeing your physician about your symptoms, how long did it take to be diagnosed with alkaptonuria?** | | |  |
| --- | --- | --- | --- |
| ⃝ ⃝ ⃝ ⃝ ⃝ ⃝ ⃝ ⃝ ⃝ Not sure |  |  | |
| D D M M Y Y Y Y | | |  |
| ⃝ Diagnosed on first visit to a physician about my symptoms | | |  |

| **How many physicians did you see before receiving your alkaptonuria diagnosis?** |
| --- |
| ⃝ ⃝ physicians (Example: 0 2) |
| ⃝ Diagnosed on first visit to a physician about my symptoms |
| ⃝ Not sure |

| **Were you initially incorrectly diagnosed with another condition?** |
| --- |
| ⃝ Yes: _________________________________________ |
| ⃝ No |
| ⃝ Not sure |

| **Do you feel that you have sufficient knowledge about alkaptonuria?** | | |
| --- | --- | --- |
| ⃝ Yes | ⃝ No | ⃝ Not sure |

| **From what sources would you say you have received the majority of information on alkaptonuria (select as many as applicable)?** | |
| --- | --- |
| ⃝ Physicians/health care professionals | ⃝ Patient organisations |
| ⃝ Literature (books/papers/scientific literature) | ⃝ Internet social forums |
| ⃝ Other | ⃝ Not sure |

| **Do you feel that your physician/health care professionals have sufficient knowledge about alkaptonuria?** | | |
| --- | --- | --- |
| ⃝ Yes | ⃝ No | ⃝ Not sure |

| **In your experience, how easy or difficult was it to…** | | | | | | |
| --- | --- | --- | --- | --- | --- | --- |
|  | **Very easy** | **Somewhat easy** | **Neither easy nor difficult** | **Somewhat difficult** | **Very difficult** | **Not sure** |
| **…communicate your symptoms to physicians or health care professionals?** |  |  |  |  |  |  |
| **…receive acknowledgement of your symptoms by physicians or health care professionals?** |  |  |  |  |  |  |
| **…receive an alkaptonuria diagnosis?** |  |  |  |  |  |  |
| **…receive adequate information about alkaptonuria?** |  |  |  |  |  |  |
| **…receive adequate treatment and care of your alkaptonuria?** |  |  |  |  |  |  |
| **…receive adequate support to manage your alkaptonuria and day-to day living?** |  |  |  |  |  |  |

| **Professionals involved in diagnosing and treating your alkaptonuria** |
| --- |


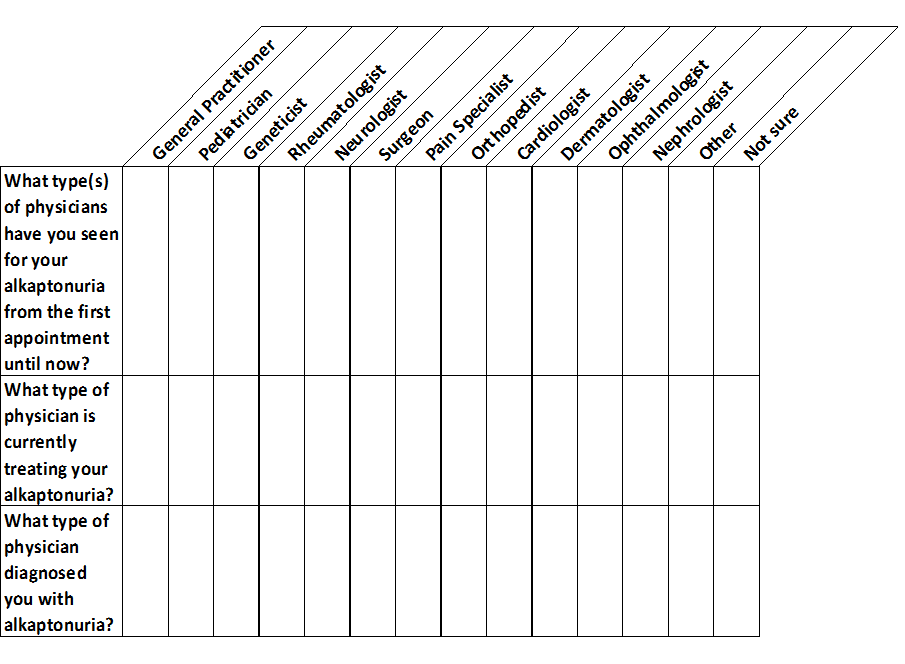

Supplement: Supplementary file 1 — Appendix S1: Supporting information [file JMD2-53-71-s001.docx]
